# Supplementary material for: Detailed molecular and epigenetic characterization of the pig IPEC-J2 and chicken SL-29 cell lines
Source: iScience. 2023 Feb 20;26(3):106252. doi: 10.1016/j.isci.2023.106252 (PMC10018572; doi:10.1016/j.isci.2023.106252)
Supplement: Data S2. Complete homer output for identified motifs in Chicken SL-29, related to Tables 5 and 6 — Homer motif analysis results for histone modifications H3K4me1, H3K4me3, H3K27ac, enhancers, and ATAC-seq of chicken SL-29 cell line. Parameters for possible false positives is as mentioned earlier for S5. [file mmc3.zip › Data_S2/S6/Chicken_SL_29/peak_files_CTCF/homerResults/motif4.similar.html]

motif4

## Information for motif4

A
T
G
C
A
T
G
C
A
C
T
G
G
T
A
C
G
T
A
C
T
G
C
A
A
C
G
T
A
T
G
C
C
A
G
T
A
G
C
T
  
Reverse Opposite:  

T
C
G
A
G
T
C
A
T
A
C
G
C
G
T
A
A
C
G
T
A
C
T
G
A
C
T
G
A
G
T
C
T
A
C
G
T
A
C
G
  

|  |  |
| --- | --- |
| p-value: | 1e-184 |
| log p-value: | -4.254e+02 |
| Information Content per bp: | 1.768 |
| Number of Target Sequences with motif | 1790.0 |
| Percentage of Target Sequences with motif | 6.07% |
| Number of Background Sequences with motif | 828.1 |
| Percentage of Background Sequences with motif | 2.85% |
| Average Position of motif in Targets | 148.0 +/- 82.0bp |
| Average Position of motif in Background | 147.9 +/- 98.4bp |
| Strand Bias (log2 ratio + to - strand density) | -0.0 |
| Multiplicity (# of sites on avg that occur together) | 1.16 |
| Motif File: | file (matrix) reverse opposite |

### Similar de novo motifs found

|  |  |  |  |  |  |  |  |
| --- | --- | --- | --- | --- | --- | --- | --- |
| Rank | Match Score | Redundant Motif | P-value | log P-value | % of Targets | % of Background | Motif file |
| 1 | 0.669 | A C T G T G C A G A T C C T A G G A C T G T A C C G T A A G C T | 1e-181 | -417.888416 | 11.96% | 7.25% | motif file (matrix) |
| 2 | 0.898 | T A G C C A T G T A G C T G A C C A T G A G T C A G T C T C G A A G C T A G T C A C G T A G C T A T C G | 1e-165 | -381.645190 | 4.02% | 1.62% | motif file (matrix) |
| 3 | 0.684 | T C A G T G A C G T A C T C A G T A G C A T G C C G T A C G A T T C G A C T G A | 1e-53 | -123.727409 | 5.54% | 3.71% | motif file (matrix) |
